# Medical Chemistry

**Published:** 1879-04

**Authors:** 


*Medical Chemistry, including the Outlines of Organic and Physiological Chemistry.* By C. GILBERT WHEELER, Professor of Chemistry in the University of Chicago. Philadelphia: Lindsay and Blakiston. Chicago: S. J. Wheeler, 1879.

The idea of dividing Chemistry into organic and animal for the benefit of physicians and medical students strikes us as appropriate and desirable. The author of this work, from long familiarity with medical men and intimate knowledge of the wants of medical students, has been able to meet the requirements of physicians and furnish a book on Medical chemistry exactly suited to their wants. The book has great practical value, is concise and comprehensive, including considerations of every subject connected with Medical chemistry, and should find its way to the library of every physician. It is *the* chemistry for a practitioner and medical student.

